# Characterization of Volatile Flavor Compounds in Dry-Rendered Beef Fat by Different Solvent-Assisted Flavor Evaporation (SAFE) Combined with GC–MS, GC–O, and OAV

**DOI:** 10.3390/foods12173162

**Published:** 2023-08-23

**Authors:** Xuelian Yang, Zhaoyang Pei, Wenbin Du, Jianchun Xie

**Affiliations:** Key Laboratory of Geriatric Nutrition and Health (Ministry of Education), Beijing Engineering and Technology Research Center of Food Additives, Beijing Technology and Business University (BTBU), Beijing 100048, China; yangxuelian@th.btbu.edu.cn (X.Y.); 2230302040@st.btbu.edu.cn (Z.P.); duwenbin@caas.cn (W.D.)

**Keywords:** beef fat, flavor, solvent-assisted flavor evaporation (SAFE), GC–MS, GC–O, OAV

## Abstract

To comprehensively understand the volatile flavor composition of dry-rendered beef fat, solvent-assisted flavor evaporation (SAFE) with four extraction solvents (dichloromethane, pentane, ethyl ether, and methanol) combined with gas chromatography–mass spectrometry (GC–MS) and gas chromatography–olfactormetry (GC–O) were performed. GC–MS analysis found 96 different volatile compounds in total using the four extraction solvents. According to the GC–MS results and the heat map and principal component analysis (PCA), most of the volatile compounds resulted from dichloromethane and pentane extraction, followed by ethyl ether. Methanol extraction found a few volatile compounds of higher polarity, which was supplementary to the analysis results. Moreover, GC–O analysis found 73 odor-active compounds in total using the four extraction solvents. The GC–O results found that pentane and dichloromethane extraction had a significantly larger number of odor-active compounds than ethyl ether and methanol extraction. This indicated that pentane and dichloromethane were more effective solvents for the extraction of odor-active compounds than the other two solvents. Finally, a total of 15 compounds of odor-active values (OAVs) ≥ 1 were determined to be the key aroma compounds in the dry-rendered beef fat, including 2–methyl–3–furanthiol, 3–methylthiopropanal, (*E*,*E*)–2,4–nonadienal, 12–methyltridecanal, and 1–octen–3–one.

## 1. Introduction

Beef is rich in B vitamins and minerals, and the protein content is up to 20% (*w*/*w*). Global bovine meat output was around 79.3 million tons in 2022, according to the Food and Agriculture Organization of the United Nations (FAO) statistical databases. Beef fat is the raw material of many meat foods, such as hot pot [1]. In particular, dry-rendered beef fat is welcome in Chinese traditional cuisine due to its unique flavor. However, its acceptance is lowered in consumers due to its high cholesterol and calories. Therefore, an investigation of the flavor composition of dry-rendered beef fat is of significance for developing a substitute for its simulated flavor.

Currently, the analysis of volatile flavor compounds in lipid matrices is mostly reported by HS–SPME combined with GC–MS. For example, Mónica Bueno et al. [2] used HS–SPME–GC–MS technology to analyze the volatile components in fresh beef with different degrees of fat oxidation, and a total of 30 volatile compounds were identified. Da et al. [3] used SPME–GC–MS technology to compare differences in volatile components of the fatty and lean parts of braised pork. Lioupi et al. [4] used HS–SPME–GC–MS technology to determine the volatile compounds in olive oil. The HS–SPME method has the advantages of short time and simple operation [5]. However, it has low extraction recovery for volatile flavor compounds in an oil or fat due to the lipophilic trait of volatile flavor compounds. Moreover, knowledge of the aroma of oil or fat is widely based on volatile compounds obtained using GC–MS rather than the odor-active compounds obtained using gas chromatography–olfactometry (GC–O) technology, though it is the odor-active compounds that actually contribute to food aroma.

Solvent-assisted flavor evaporation (SAFE) is known to be able to extract volatile fractions with a high recovery yield from the food matrix. It can avoid potential flavor alteration or pseudo-product formation as it works under high vacuum and mild temperatures. In particular, SAFE is versatile in its selection of extraction solvents to isolate the target volatile components from a lipid matrix. Gerlach et al. [6] used methanol as the extraction solvent for SAFE/GC–MS and GC–O analysis of the aroma components in pig fat, and 16 compounds such as 2,4–heptadienal, nonanal, and (*E*,*E*)–2,4–decadienal were determined as key aroma compounds. Zhou et al. [7] used dichloromethane as the extraction solvent during a SAFE/GC–MS analysis of aroma components in olive oil, while 54 volatile compounds, including aldehydes, ketones, acids, and esters, were identified. 

In this study, the SAFE treatment by four extraction solvents, namely, dichloromethane, pentane, ethyl ether, and methanol, combined with GC–MS and GC–O, were performed to analyze aroma compounds in dry-rendered beef fat. The effects of these solvents on the analysis results were evaluated while the better ones were selected for quantitative analysis and odor-active values (OAVs) calculation to expose the key aroma compounds. The research results can provide guidance for the preparation of flavorings of dry-rendered beef fat flavor and improvable utilization of beef fat in the food industry.

## 2. Materials and Methods

### 2.1. Materials and Reagents

Abdominal subcutaneous fat of 24 month old Inner Mongolia yellow beef was bought from a supermarket in Beijing, China. The acid value of the fat was determined to be 1.63 (mgKOH/g), according to the National Standard GB5009.229–2016. Before use, the subcutaneous fat was stored at −20 °C.

The following substances, including 1,2–dichlorobenzene (99%), butyraldehyde (95%), 3–methylbutanal (98%), 2–methylbutyraldehyde (95%), hexanal (98%), heptanal (99%), (*E*)–2–hexenal (95%), octanal (95%), nonanal (99%), (*E*)–2–nonenal (99%), (*E*)–2–octenal (95%), (*E*,*E*)–2,4–octadieneal (99%), (*E*,*E*)–2,4–heptadienal (99%), benzaldehyde (95%), (*E*,*E*)–2,6–nonadienal (99%), 2–decenal (99%), (*E*,*E*)–2,4–nonadienal (99%), decanal (99%), (*E*,*E*)–2,4–decadienal (98%), 2–dodecenal (95%), (*E*)–2–undecenal (99%), 2–pentanol (99%), 1–octen–3–ol (99%), 1–heptanol (99%), 1–hexanol (95%), 1–octanol (98%), 2,3–butanediol (95%), (*E*)–2–octen–1–ol (99%), phenylethyl alcohol (99%), 1–undecanol (99%), 1–dodecanol (99%), 1,4–pentanediol (99%), (*Z*)–4–dodecanol (99%), hexanone (99%), 2–nonanone (99%), 1–octen–3–one (99%), 2–undecanone (99%), 2–tetradecanone (99%), 2–heptadecanone (99%), acetic acid (99%), propionic acid (99%), 2–methylpropionic acid (99%), butyric acid (99%), 4–hydroxybutyric acid (99%), 3–methylbutyric acid (99%), valeric acid (99%), heptanoic acid (99%), nonanoic acid (99%), benzoic acid (95%), dimethyl disulfide (99%), diethyl disulfide (99%), 2–methyl–3–furanthiol (95%), 12–methyltridecanal (99%), 2–mercaptothiophene (99%), 3–methylthiopropanal (99%), thiophene (95%), 2–pentylthiophene (99%), thiazole (99%), 4–methylthiazole (99%), 4–methyl–5–thiazolylethanol (95%), 2,5–dimethylpyrazine (99%), 2,3–dimethylpyrazine (99%), 2–ethyl–3,5–dimethylpyrazine (99%), 2–acetylpyrazine (99%), 2–ethyl–6–methylpyrazine (99%), trimethylpyrazine (99%), 2–acetyl–3–methylpyrazine (99%), 2–pentylfuran (99%), furfural (99%), *p*–cresol (99%), 3–methylphenol (99%), vanillin (99%), 4–ethylphenol (99%), 2,5–dimethyl–4–hydroxy–3(2*H*)–furanone(99%), and *γ*–octalactone (99%), were all bought from *J&K* Chemical Ltd. (Beijing, China). Fatty acid methyl ester (FAME) standards were purchased from ANPEL Laboratory Technologies Inc. (Shanghai, China). Other chemicals used in this study were all commercially obtained and of analytical grade.

### 2.2. Dry-Rendered Beef Fat Preparation

The beef fat was first minced into about 1 × 1 × 1 cm^3^ cubes and then heated at 130 ± 5 °C for 20 min with an induction cooker while manually stirring. Three replicates were performed. 

The color values of the dry-rendered beef fat were L* = 80.39, a* = 2.04, and b* = 16.27, measured using a colorimeter (UltraScan PRO, Hunter Associates Laboratory, Inc., Reston, VA, USA). The acid value was 1.92 (mgKOH/g), determined according to the National Standard GB5009.229–2016. The prepared dry-rendered beef fat was stored at −20 °C before analysis.

### 2.3. Fatty Acid Analysis

First, the raw subcutaneous beef fat was homogenized and extracted using dichloromethane and methanol (2:1, *v*/*v*) to obtain the fat fraction. Then, the dry-rendered fat and the fat fraction were methyl esterified and analyzed by GC–MS, respectively, as described in our previous work [8]. Briefly, an Agilent 7890A/5975B GC–MS system with a HP–5 capillary column (30 m × 0.25 mm × 0.25 μm) (Agilent Technologies, Santa Clara, CA, USA) was used. The fatty acid methyl esters were identified by the search through the NIST 11 mass spectra database and injection of the standards of fatty acid esters. The results were expressed as percentages (%) of total fatty acids, which were derived via normalization of peak areas of the fatty acids esters. Three replicates were performed.

### 2.4. SAFE Extraction

The solvents dichloromethane, n–pentane, ethyl ether, and methanol were purified by distillation. An aliquot of 500 g of the dry-rendered beef fat was extracted using 500 mL of each solvent three times (500 mL × 3). The extract was combined and then subject to SAFE treatment as described by Zhao et al., 2017 [9]. The temperature of the water bath with a sample flask was 30 °C, the circulating water temperature was 50 °C, and the system pressure was less than 10^−5^ Pa. The received distillate was dried with anhydrous sodium sulfate. Then, it was first concentrated to 5 mL using a Vigreux column (I.D. 50 cm × 1 cm) and then to 0.3 mL using a stream of gentle nitrogen gas.

### 2.5. GC–MS Analysis

The 7890A–5975C gas chromatography–mass spectrometry (Agilent, Santa Clara, CA, USA) with a DB–WAX (30 m × 0.25 mm × 0.25 μm) column was used. The column temperature was initially 40 °C, then it was raised at 2.5 °C/min to 200 °C, and finally raised at 6 °C/min to 240 °C. The carrier gas was He (99.999%) at 1 mL/min. The sample of 1 μL was injected at 250 °C at a split ratio of 20:1.

The mass detector was operated at 150 °C in electron impact mode at 70 eV. The ion source temperature was 230 °C. The transfer line temperature was 250 °C. The chromatograms were recorded by monitoring the total ion current in the 40~450 mass range.

Compounds were identified based on the NIST11 library, retention index, and authentic chemicals injection. The amount of volatile compounds was derived via the normalization of peak areas of the detected compounds.

### 2.6. GC–O Analysis

As described by Zhao et al., 2017 [9], an Agilent 7890A gas chromatograph (Agilent Technologies, Santa Clara, CA, USA) equipped with an FID detector and a DATU 2000 high-resolution olfactometer (DATU Inc., New York, NY, USA) was used. The capillary column was DB–WAX (30 m × 0.25 mm × 0.25 μm). The column temperature was initially 40 °C and then was raised to 230 °C at 5 °C/min. The carrier gas was N_2_ (purity of 99.999%) in the flow rate of 1 mL/min. The sample of 1 μL was injected at 250 °C in a splitless mode.

A panel of twelve graduate students were trained prior to GC–O analysis. The odor descriptors were determined through the discussion of the panelists. During the GC–O analysis, each panelist recorded the sniffed odors with retention times, which were then converted to RI values relative to the *n*–alkane series (C_5_~C_29_). The results were expressed as NIF (%)—that is, a percentage of the summed frequencies of a compound perceived by all panelists divided by the total times of a sample analyzed.

The compounds were identified based on the GC–MS identification results, the sniffed retention indices and odor characteristics, and the injection of authentic chemicals.

### 2.7. Quantitation of Odor-Active Compounds

The GC–MS instrument and analytical conditions used were as same as those described in the above section on GC–MS analysis, whereas the selective ion monitoring mode (SIM) was adopted. The analyzed samples were the concentrates after SAFE treatment using pentane and dichloromethane as the solvents. The ratio of the peak area of compound to that of the internal standard 1,2–dichlorobenzene (30 μg/mL) was used in the calculation of calibration curves. The amounts of compounds were expressed as ng/g in the dry-rendered beef fat.

### 2.8. Statistical Analysis

The results were means of three replicates. ANOVA was used to identify significant difference (*p* < 0.05) among the means. Tables were drawn using Microsoft Excel 2016 software. Principal component analysis (PCA) was performed using SIMCA13.0 (Version 13.0, Umetrics AB, Umeå, Sweden). Heat maps were plotted using Heml1.0 (Huazhong University of Science and Technology, Wuhan, China).

## 3. Results and Discussion

### 3.1. Fatty Acid Composition

As shown in Table 1, a total of 12 fatty acids were found in the raw beef fat, while oleic acid (C18:1), palmitic acid (C16:0), and stearic acid (C18:0) were the major fatty acids. This agreed with the reports of Onopiuk et al. [10] on beef fat fatty acid composition analysis. Compared to the raw beef fat, in the dry-rendered beef fat, the content of polyunsaturated fatty acid (PUFA) of C18:2 was decreased significantly. This was because the PUFAs were apt to undergo lipid oxidization and degradation during the heating processing, which could produce volatile flavor compounds [11,12].

### 3.2. Volatile Compounds Identified Using GC–MS

In order to comprehensively analyze the volatile compounds, four solvents, namely dichloromethane, pentane, ethyl ether, and methanol were used, respectively, in the SAFE treatment. As shown in Table 2, a total of 96 volatile compounds were identified by GC–MS analysis, including 19 aldehydes, 16 alcohols, 9 ketones, 21 acids, 6 lactones, 9 sulfur-containing compounds, 8 nitrogen-containing heterocyclic compounds, 5 oxygen-containing heterocyclic compounds, and 3 others. Among them, (*E*)–octenal, (*E*)–nonenal, pentadecanoic, and 2–undecanone were found in cooked beef cuts [13]. 2,5–Dimethylpyrazine and furfural were found in grilled beef striploins [14]. Heptanal, hexanal, nonanal, (*E*,*E*)–2,4–nonadienal, 1–octen–3–ol, 1–heptanol, 1–hexanol, 1–octanol, 2,3–butanediol, and 2–decanone were detected in beef tallow [15]. 2–Pentylfuran was reported in roasted beef [16].

In comparison, most volatile compounds could be identified using three solvents, namely dichloromethane, pentane, and ethyl ether, for the SAFE/GC–MS analysis. Using methanol as the extraction solvent only found a small number of compounds because of its high polarity. Nonetheless, with methanol extraction, eight volatile compounds were identified as a supplement to those found in the other three solvents, namely acetaldehyde, 3–hydroxybutyraldehyde, 3–hexanol, 4–pentenoic acid, heptadecanic acid, 2,3–dihydroxy–5–methylthiophene, 2,5–dimethylfuran, and 3–methylphenol. Taken together with the GC–MS results of the four solvents, it showed that aldehydes accounted for the highest proportion of total volatile compounds (31~39%), followed by acid compounds (26~32%). Acetic acid, hexanoic acid, (*E*)–2–nonenal, nonanal, octanal, 3–ethyl–2,5–dimethylpyrazine, acetoin, (*E*)–2–undecenal, 2,5–dimethylpyrazine, etc., had high contents (≥2.6%) using the three solvents (dichloromethane, pentane, and ethyl ether).

In Table 2, the detected sulfur-containing compounds, nitrogen-containing heterocyclic compounds, and oxygen-containing heterocyclic compounds were mainly produced via the Maillard reaction. Usually, the sulfur-containing compounds have the characteristics of a meaty aroma, and the nitrogen-containing compounds (pyrazine) have the characteristics of a roasted aroma. These compounds play important roles in cooked meat flavor due to their low odor thresholds. 

The detected aldehydes (e.g., nonanal, hexanal, heptanal, (*E*)–2–undecanal, (*E*,*E*)–2,4–decadienal, (*E*,*E*)–2,4–heptadienal) and alcohols (e.g., 1–octen–3–ol) were all of lipid oxidization and degradation compounds. Among them, octanal, decanal, and nonanal could be derived from the oxidative degradation of oleic acid. Hexanal, (*E*,*E*)–2,4–heptadienal, (*E*,*E*)–2,4–decadienal, 1–octen–3–ol, and 2–pentylfuran could be formed from the oxidative degradation of linoleic acid. In livestock and poultry meat, oleic acid is the most common monounsaturated fatty acid, while linoleic acid is the most common polyunsaturated fatty acid [17]. Among the compounds in Table 2, hexanal, heptanal, and (*E*,*E*)–2,4–decadienal were found in the surimi of catfish [18]. Hexanal, heptanal, octanal, nonanal, and 1–octen–3–ol were detected in Beijing roast duck [19]. Octanal and nonanal were detected in grilled mutton shashlik [12]. (*E*)–2–decenal,1–octen–3–ol, and hexanal were found in stewed goat meat [20].

**Figure 1:**
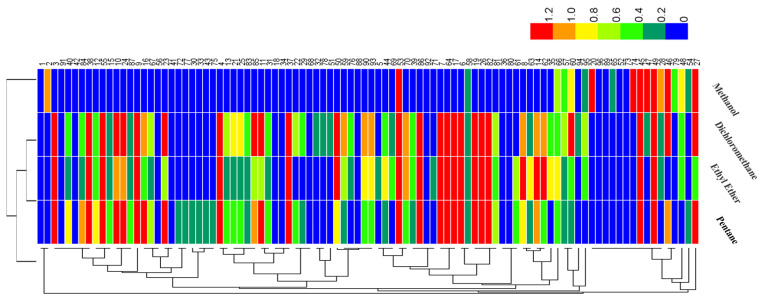
Heat map of volatile compounds identified in the dry-rendered beef fat using four extraction solvents (pentane, ethyl ether, dichloromethane, and methanol) for solvent-assisted flavor evaporation combined with gas chromatography–mass spectrometry (SAFE/GC−MS) analysis.

**Figure 2:**
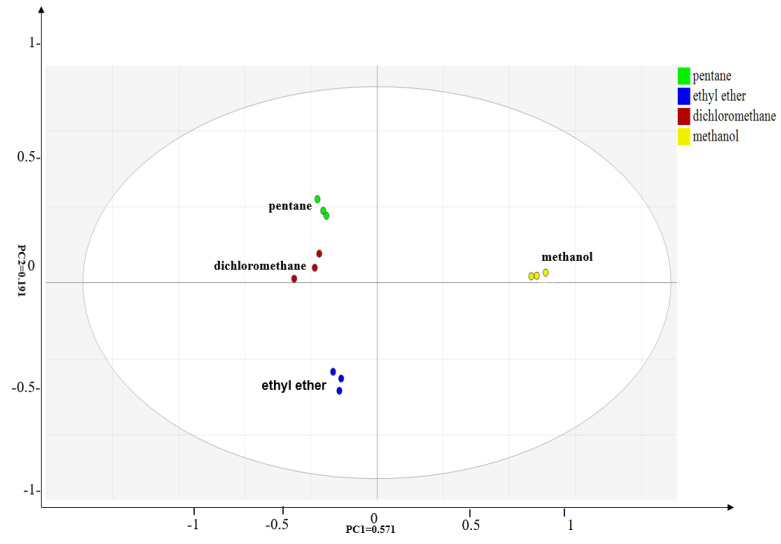
Score plot of volatile compounds in the dry-rendered beef fat using four extraction solvents (pentane, ethyl ether, dichloromethane, and methanol) for solvent-assisted flavor evaporation combined with gas chromatography–mass spectrometry (SAFE/GC−MS) analysis.

Moreover, GC–MS results (Table 2) were processed via heat map and principal component analysis (PCA), as shown in Figure 1 and Figure 2, respectively. As illustrated in the legend (Figure 1), the colors of the block from red to blue indicated that the compounds were present from higher to lower levels. It could be seen from Figure 1 that the volatile components found by SAFE/GC–MS using dichloromethane, pentane, and ethyl ether as the extraction solvents were similar, which had a markedly greater number of identifications than by methanol extraction. Methanol only extracted a small number of compounds due to its high polarity. Figure 2 shows that the contribution rate of the first principal component was 57.1%, the second principal component was 19.1%, and the cumulative contribution rate was 76.2%, indicating that the PCA analysis represented most of the information of the original variables and the analysis results were reliable. According to the score plot (Figure 2), the samples of pentane, ethyl ether, and dichloromethane were distributed closer, indicating that volatile composition extracted using the three solvents had a high similarity. In comparison, the position of the sample point for methanol extraction was far from the position of the points for pentane, ethyl ether, and dichloromethane extraction, which demonstrated the significant difference in extraction effects between them.

**Figure 3:**
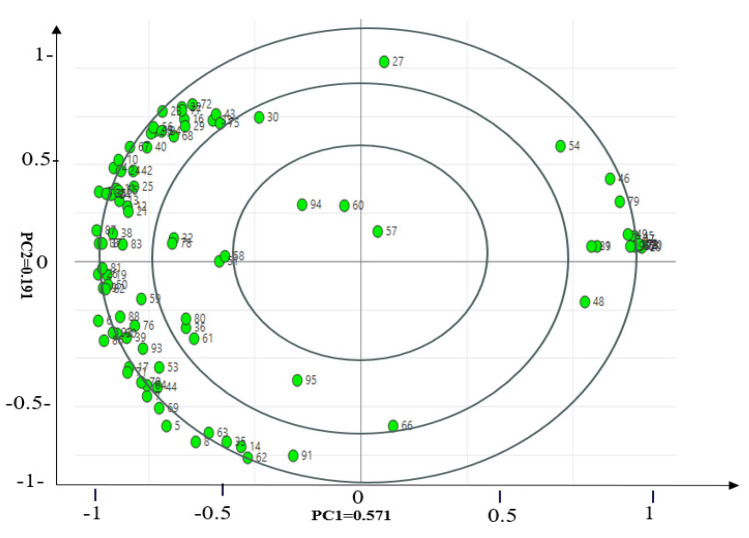
Loading plot of volatile compounds in the dry-rendered beef fat using four extraction solvents (pentane, ethyl ether, dichloromethane, and methanol) for solvent-assisted flavor evaporation combined with gas chromatography−mass spectrometry (SAFE/GC−MS) during the principal component analysis (PCA) analysis.

It could be seen from Figure 3 that the position of the most volatile compounds was well correlated with the position of dichloromethane and pentane on the score plot of Figure 2. This indicated that dichloromethane and pentane extraction had a strong positive correlation with the most volatile compounds. Moreover, the position of ethyl ether in Figure 2 also showed as being somewhat well correlated with the most volatile compounds in Figure 3, indicating that ethyl ether extraction also had certain positive correlation. In other words, the aforementioned three solvents displayed good performance in the extraction of the most volatile compounds. However, methanol was not a good extraction solvent, because it was far away from the most volatile compounds on the loading plot (Figure 3). The above results were consistent with the above heat map analysis and GC−MS analysis results.

### 3.3. Odor-Active Compounds Identified by GC–O

Unlike GC–MS, GC–O focuses on screening the odor-active compounds (i.e., odorants) in food that actually contribute to food aroma. In the SAFE/GC–O analysis, the four solvents of pentane, ethyl ether, dichloromethane, and methanol were also utilized in this study. As shown in Table 3, in total, 80 odorants were identified, including 26 aldehydes, 9 alcohols, 8 ketones, 11 sulfur-containing compounds, 5 nitrogen-containing heterocyclic compounds, 5 acids, 1 oxygen-containing heterocyclic compound, 2 lactones, and 13 other compounds. The involved odors mainly included meaty, green, fatty, roasted, and creamy aromas, and so on. As for frequency detection of GC–O analysis, the higher the NIF value, the greater the contribution to the overall aroma. In Table 3, 33 compounds with high NIF values (≥80%) were extracted by the four solvents, including 3–methylbutanal, hexanal, octanal, (*E*,*Z*)–2,4–heptadienal, (*E*,*E*)–2,6–nonadienal, (*E*,*E*)–2,4–decadienal, 2–pentanol, 1–octen–3–one, 3–methylthiopropanal, 4–methylthiazole, 2–acetylthiazole, 2,5–dimethylpyrazine, 2–ethyl–3,5–dimethylpyrazine, 4–hydroxybutyric acid, 4–hydroxybutyric acid, *p*–cresol, etc. Therefore, these compounds might contribute greatly to the aroma of the dry-rendered beef fat. Remarkably, 3–methylbutanal (malty) was reported to play an important role in beef flavor [21]. Pyrazine compounds, such as trimethylpyrazine, are known as important contributors to the roasted smell of cooked beef [17]. Among the aforementioned compounds, hexanal and (*E*,*E*)–2,4–decadienal were found in stewed beef [22] and stewed black pork broth [9]. Octanal, 2–ethyl–3,5–dimethylpyrazine, and *p*–cresol were found in beef tallow residue hydrolysate [23]. 

In agreement with the GC–MS results, similar numbers of odorants were found in the SAFE/GC–O analysis using the three different solvents (pentane, dichloromethane, and ethyl ether) for the extraction, namely 45, 40, and 39, respectively. Only 28 odorants were extracted using methanol due to its high polarity. Moreover, the SAFE/GC–O analysis using pentane extraction had 24 compounds of NIF ≥ 80%, including 3–methylbutanal, hexanal, (*E*,*Z*)–2,4–heptadienal, (*E*,*E*)–2,6–nonadienal, 3–methylthiopropanal, 2–acetylthiazoline, 2–pentanol, 1–octen–3–one, 4–hydroxybutyric acid and *p*–cresol, etc. The SAFE/GC–O analysis using dichloromethane extraction had 12 compounds of NIF ≥ 80%, including (*E*,*E*)–2,4–decadienal, 4–methylthiazole, 2,5–dimethylpyrazine, 2–ethyl–3,5–dimethylpyrazine, etc. The SAFE/GC–O analysis by ethyl ether extraction had 10 compounds of NIFs ≥ 80%, including octanal, (*E*,*E*)–2,6–nonadienal, 3–methylthiopropanal, 2,5–dimethyl–4–hydroxy–3(2*H*)–furanone, *p*–cresol, etc. The SAFE/GC–O analysis using methanol extraction only had three compounds of NIF ≥ 80%, which were 3–methylthiopropanal, 2,5–dimethylpyrazine, and 2,5–dimethyl–4–hydroxy–3(2*H*)–furanone.

**Figure 4:**
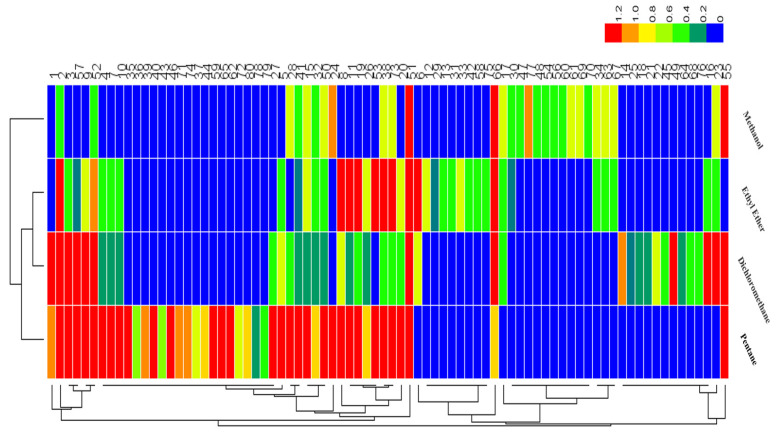
Heat map of odor-active compounds identified in the dry-rendered beef fat using four extraction solvents (pentane, ethyl ether, dichloromethane, and methanol) for solvent-assisted flavor evaporation combined with gas chromatography–olfactometry (SAFE/GC−O) analysis.

Heat map analysis of the odorants was carried out, as shown in Figure 4, where the colors of blocks from red to blue indicated the NIF values ranged from higher to lower levels. Combined with GC–O results (Table 3), this analysis showed that pentane and dichloromethane had better performance for odorants extraction. Ethyl ether as the extraction solvent was not as good, since a smaller number of compounds were detected with high detection frequency (NIF ≥ 80%). Methanol as the extraction solvent was poor, since few odor-active compounds with high detection frequency (NIF ≥ 80%) were found. Furthermore, it could be noted that those odorants found through ethyl ether and methanol extraction were likewise able to be found using pentane and dichloromethane extraction. Therefore, pentane and dichloromethane were selected to be extraction solvents for the quantitative analysis.

### 3.4. Quantitation and OAV Calculation

Normally, potential higher-contribution compounds, screened using GC–O, are considered as key aroma compounds when their odor-active values (OAVs) exceed or are equal to one (OAVs ≥ 1). Therefore, those compounds mentioned above of NIFs ≥ 80% are mainly quantitated via GC–MS in SIM detection mode (Table 4).

As shown in Table 5, when using dichloromethane as the extraction solvent for the SAFE/GC–MS quantitation, 15 compounds had OAVs ≥ 1, including 2–methyl–3–furanthiol, 3–methylthiopropanal, (*E*,*E*)–2,4–nonadienal, 12–methyltridecanal, 1–octen–3–one, 2–pentylfuran, and so on. Among them, 12–Methyltridecanal is known as a species-specific odorant of stewed beef [24]. When using pentane as the extraction solvent for the SAFE/GC–MS quantitation, 11 compounds were found with OAVs ≥ 1, which were 2–methyl–3–furanthiol, 3–methylthiopropanal, 12–methyltridecanal, (*E*,*E*)–2,4–nonadienal, 2–decenal, 1–octen–3–one, etc. Overall, 2–methyl–3–furanthiol (meaty) and 3–methylthiopropanal (malty) had high OAVs by both extraction solvents, of which 2–methyl–3–furanthiol is known as a meaty flavor in cooked meat [17].

Checking the literature on meat flavor analysis, 3–methylthiopropanal and 4–ethylphenol were found as key aroma compounds in grilled mutton shashlik by Du et al. [12]. 2–Methyl–3–furanthiol and (*E*,*E*)–2,4–decadienal were found as key aroma compounds in stewed chicken broth by Fan et al. [25]. Hexanal, (*E*,*E*)–2,4–nonadienal, (*E*,*E*)–2,4–decadienal, and *p*–cresol were found as key aroma compounds in Tibetan dzo beef meat by Wan et al. [22].

## 4. Conclusions

SAFE treatment using four different polarity solvents (dichloromethane, pentane, ethyl ether, and methanol) was performed for the analysis of volatile favors of the dry–rendered beef fat. GC–MS analysis found, in total, 96 different volatile compounds using the four extraction solvents, among which the aliphatic aldehydes from lipid degradation had the highest amounts. Comparatively, dichloromethane and pentane as the extraction solvents demonstrated the best performance, followed by ethyl ether, while methanol as the extraction solvent provided extra information on some polar volatile compounds. On the other hand, GC–O analysis found, in total, 73 different odorants using the four extraction solvents, while 33 compounds displayed powerful odor activities (NIF ≥ 80%). During the GC–O analysis, both dichloromethane and pentane as the extraction solvents resulted in a complete and greater number of odorants found. Therefore, quantitation was conducted using both of them as the extraction solvents for the SAFE treatment. Finally, OAV calculation showed, in total, 15 compounds of OAV ≥ 1, including 2–methyl–3–furanthiol, 3–methylthiopropanal, (*E*,*E*)–2,4–nonadienal, 12–methyltridecanal, 1–octen–3–one, etc., which were considered to be the key aroma compounds in the dry-rendered beef fat. In the future, aroma recombination will be conducted to further confirm the identification results. In addition, we will prepare flavorings to simulate the dry-rendered beef fat flavor.

## Figures and Tables

**Table 1:** Compositions of fatty acid in the raw beef fat and the dry-rendered beef fat.

Fatty Acids ^1^	Contents ^2^ (%)	
	Raw Beef Fat	Dry-Rendered Beef Fat
C12:0	0.41 ± 0.00 a	0.10 ± 0.01 b
C14:0	0.30 ± 0.01 b	3.97 ± 0.26 a
C16:0	27.86 ± 1.08 a	26.08 ± 0.52 a
C18:0	27.68 ± 0.20 a	22.23 ± 0.38 b
C20:0	0.44 ± 0.06 a	0.19 ± 0.01 b
C22:0	0.11 ± 0.03	–
SFA	56.80	52.57
C14:1	0.59 ± 0.02 b	1.11 ± 0.07 a
C16:1	0.63 ± 0.01 b	5.92 ± 0.40 a
C18:1	37.14 ± 1.24 a	38.60 ± 1.72 a
C20:1	0.41 ± 0.01 a	0.20 ± 0.00 b
MUFA	38.77	45.83
C18:2	4.43 ± 0.1 a	0.42 ± 0.00 b
C18:2tt	–	1.18 ± 0.06
PUEA	4.43	1.60

^1^ SFA, saturated fatty acid; MUFA, monounsaturated fatty acid; PUFA, polyunsaturated fatty acid. ^2^ Means ± SD (n = 3); means with different letters in the same row indicate significant differences (*p* < 0.05), –, not detected.

**Table 2:** Volatile compounds identified in the dry-rendered beef fat using four extraction solvents (pentane, ethyl ether, dichloromethane, and methanol) for solvent-assisted flavor evaporation combined with gas chromatography–mass spectrometry (SAFE/GC–MS) analysis.

No.	RIs ^1^	Compounds	Relative Peak Area (%) ^2^				Identification Methods ^3^
			Pentane	Ethyl Ether	Dichloromethane	Methanol	
		Aldehydes (19)					
1	692	Acetaldehyde	–	–	–	0.03 ± 0.12	RI/MS
2	812	3–Hydroxybutyral	–	–	–	0.96 ± 0.12	RI/MS
3	1078	Hexanal	3.68 ± 0.74 a	2.01 ± 0.19 b	3.31 ± 0.46 a	–	RI/MS/S
4	1174	Heptanal	5.45 ± 1.09 a	2.41 ± 0.23 c	3.91 ± 0.55 b	–	RI/MS/S
5	1319	(*E*)–2–Hexenal	0.06 ± 0.01 c	0.20 ± 0.02 a	0.12 ± 0.02 b	–	RI/MS/S
6	1272	Octanal	3.67 ± 0.73 b	4.25 ± 0.41 a	2.98 ± 0.42 b	–	RI/MS/S
7	1370	Nonanal	3.75 ± 0.75 b	7.09 ± 0.69 a	3.29 ± 0.46 b	–	RI/MS/S
8	1401	(*Z*)–2–Nonenal	0.76 ± 0.15 b	2.97 ± 0.29 a	1.14 ± 0.16 b	–	RI/MS
9	1403	(*E*)–2–Nonenal	4.26 ± 0.85 a	4.03 ± 0.39 a	2.73 ± 0.38 b	–	RI/MS/S
10	1422	(*E*)–2–Octenal	2.50 ± 0.50 a	0.96 ± 0.09 b	2.27 ± 0.32 a	–	RI/MS/S
11	1484	(*E*,*E*)–2,4–Heptadienal	1.40 ± 0.36 a	0.70 ± 0.17 b	1.47 ± 0.39 a	–	RI/MS/S
12	1486	Benzaldehyde	0.90 ± 0.23 a	0.52 ± 0.13 b	0.50 ± 0.13 b	–	RI/MS/S
13	1595	(*E*,*E*)–2,6–Nonadienal	0.48 ± 0.12 a	0.26 ± 0.06 b	0.60 ± 0.16 a	–	RI/MS/S
14	1632	(*E*)–2–Decenal	1.09 ± 0.28 c	5.39 ± 1.31 a	0.98 ± 0.26 b	–	RI/MS/S
15	1664	(*E*,*E*)–2,4–Nonadienal	1.41 ± 0.37 a	0.26 ± 0.06 b	1.29 ± 0.34 a	–	RI/MS/S
16	1680	Decanal	2.38 ± 0.62 a	0.39 ± 0.09 c	1.05 ± 0.28 b	–	RI/MS/S
17	1717	(*E*)–2–Undecenal	2.60 ± 0.68 b	4.19 ± 1.02 a	2.64 ± 0.70 b	–	RI/MS
18	1729	Dodecanal	0.07 ± 0.02 a	0.05 ± 0.01 a	0.08 ± 0.02 a	–	RI/MS
19	1774	(*E*,*E*)–2,4–Decadienal	2.30 ± 0.60 b	2.39 ± 0.58 b	3.52 ± 0.23 a	–	RI/MS/S
		Subtotal	36.75	38.09	31.88	1.00	
		Alcohols (16)					
20	1211	3–Hexanol	–	–	–	4.18 ± 0.55	RI/MS
21	1237	2–Pentanol	0.51 ± 0.13 b	0.30 ± 0.07 c	0.78 ± 0.21 a	–	RI/MS/S
22	1411	2–Octanol	0.50 ± 0.16 a	0.01 ± 0.00 b	0.58 ± 0.16 a	–	RI/MS
23	1423	1–Octen–3–ol	3.65 ± 1.18 a	0.69 ± 0.16 c	2.70 ± 0.74 b	–	RI/MS/S
24	1437	1–Heptanol	2.18 ± 0.70 a	1.10 ± 0.26 b	1.63 ± 0.45 a	–	RI/MS/S
25	1454	1–Hexanol	0.53 ± 0.17 b	0.30 ± 0.07 b	0.84 ± 0.23 a	–	RI/MS/S
26	1538	Octanol	3.17 ± 1.03 a	3.32 ± 0.78 a	3.49 ± 0.95 a	–	RI/MS/S
27	1581	2,3–Butanediol	2.71 ± 0.88 a	0.50 ± 0.12 b	1.68 ± 0.46 a	1.76 ± 0.52 a	RI/MS/S
28	1600	1,2–Propanediol	0.17 ± 0.05 a	0.20 ± 0.05 b	0.26 ± 0.07 b	1.07 ± 0.10 a	RI/MS
29	1642	(*E*)–2–Octen–1–ol	0.26 ± 0.08 a	0.01 ± 0.00 b	0.40 ± 0.11 a	–	RI/MS/S
30	1673	1–Nonanol	0.25 ± 0.08 a	0.02 ± 0.00 b	–	–	RI/MS
31	1843	Phenylethyl alcohol	0.47 ± 0.15 a	0.30 ± 0.07 b	0.49 ± 0.13 a	–	RI/MS/S
32	1879	1–Undecanol	0.07 ± 0.02 b	0.07 ± 0.02 b	0.23 ± 0.06 a	–	RI/MS/S
33	1964	1–Dodecanol	0.21 ± 0.07 a	0.04 ± 0.01 b	0.05 ± 0.01 b	–	RI/MS/S
34	2054	1,4–Pentadiol	0.04 ± 0.01 a	0.03 ± 0.01 a	0.05 ± 0.01 a	–	RI/MS/S
35	2150	2–Hexyl–1–dodecanol	0.07 ± 0.01 c	0.88 ± 0.24 a	0.40 ± 0.15 b	–	RI/MS
		Subtotal	14.80	7.76	13.61	7.00	
		Ketones (9)					
36	1197	1–Octanone	0.14 ± 0.03 a	0.16 ± 0.04 a	0.04 ± 0.01 b	–	RI/MS
37	1262	Acetoin	3.12 ± 0.63 a	2.60 ± 0.70 a	3.11 ± 1.14 a	–	RI/MS
38	1307	2,3–Octanedione	2.53 ± 0.52 a	1.88 ± 0.51 b	1.68 ± 0.62 b	–	RI/MS
39	1494	2–Decanone	0.32 ± 0.07 b	0.53 ± 0.14 a	0.49 ± 0.18 a	–	RI/MS
40	1599	2–Undecanone	0.80 ± 0.16 a	0.30 ± 0.08 b	0.42 ± 0.15 b	–	RI/MS/S
41	1709	2–Dodecanone	0.12 ± 0.02 a	0.03 ± 0.01 b	0.12 ± 0.05 a	–	RI/MS
42	1816	2–Tridecanone	0.15 ± 0.03 a	0.07 ± 0.02 b	0.09 ± 0.03 b	–	RI/MS
43	1914	2–Tetradecenone	0.36 ± 0.07 a	0.05 ± 0.01 b	0.08 ± 0.03 b	–	RI/MS/S
44	2195	2–Heptadecanone	0.34 ± 0.07 c	0.80 ± 0.22 a	0.54 ± 0.20 b	–	RI/MS/S
		Subtotal	7.90	6.41	6.55	–	
		Acids (21)					
45	1429	Acetic acid	8.40 ± 1.71 b	5.70 ± 1.54 c	8.27 ± 3.03 b	70.01 ± 21 a	RI/MS/S
46	1508	Propionic acid	1.37 ± 0.28 b	0.13 ± 0.03 c	1.26 ± 0.46 b	4.16 ± 1.25 a	RI/MS/S
47	1581	2–Methylpropionic acid	0.11 ± 0.02 c	0.05 ± 0.01 c	0.21 ± 0.08 b	3.18 ± 0.96 a	RI/MS/S
48	1637	Butyric acid	0.37 ± 0.07 c	0.51 ± 0.14 b	0.42 ± 0.15 c	0.79 ± 0.24 a	RI/MS/S
49	1640	4–Hydroxybutyric acid	1.55 ± 0.37 b	1.32 ± 0.38 b	1.25 ± 0.30 b	5.23 ± 1.57 a	RI/MS/S
50	1680	3–Methylbutyric acid	0.97 ± 0.23 b	1.22 ± 0.35 a	1.53 ± 0.37 a	–	RI/MS/S
51	1760	Pentanoic acid	0.06 ± 0.01 c	0.11 ± 0.03 b	0.30 ± 0.07 a	0.05 ± 0.01 c	RI/MS/S
52	1770	4–Pentenoic acid	–	–	–	0.06 ± 0.02	RI/MS
53	1854	Hexanoic acid	4.98 ± 1.18 b	8.97 ± 2.60 a	7.29 ± 1.75 a	2.30 ± 0.69 c	RI/MS/S
54	1961	Heptanoic acid	0.26 ± 0.06 a	0.08 ± 0.02 b	0.06 ± 0.01 c	0.33 ± 0.1 a	RI/MS/S
55	2066	Octanoic acid	3.83 ± 0.91 a	2.25 ± 0.65 b	2.30 ± 0.55 b	–	RI/MS/S
56	2153	Nonanoic acid	0.08 ± 0.02 a	0.02 ± 0.01 c	0.05 ± 0.01 b	–	RI/MS/S
57	2342	(*E*)–9–Decenoic acid	0.30 ± 0.07 c	0.33 ± 0.10 c	0.61 ± 0.15 a	0.44 ± 0.11 b	RI/MS
58	2422	Benzoic acid	0.36 ± 0.08 a	0.36 ± 0.10 a	0.34 ± 0.08 a	0.26 ± 0.06 b	RI/MS/S
59	2448	Dodecanoic acid	0.35 ± 0.08 c	0.61 ± 0.18 b	0.95 ± 0.23 a	–	RI/MS
60	2490	Decanoic acid	0.44 ± 0.10 c	0.37 ± 0.11 c	1.39 ± 0.33 a	0.74 ± 0.19 b	RI/MS
61	2511	Oleic acid	0.45 ± 0.11 a	0.55 ± 0.13 a	0.10 ± 0.02 b	–	RI/MS
62	2692	Myristic acid	0.44 ± 0.11 c	4.46 ± 1.08 a	1.07 ± 0.26 b	–	RI/MS
63	2821	Pentadecanoic acid	0.27 ± 0.07 b	0.90 ± 0.22 a	0.29 ± 0.07 b	–	RI/MS
64	2850	Lactic acid	2.00 ± 0.51 b	3.61 ± 0.88 a	1.98 ± 0.47 b	–	RI/MS
65	2885	Heptadecanoic acid	–	–	–	0.29 ± 0.07	RI/MS
		Subtotal	26.57	31.53	29.64	87.85	
		Lactones (6)					
66	1617	4–Hydroxy–5–methyl–2(3*H*)–furanone	0.47 ± 0.12 b	0.80 ± 0.19 a	0.50 ± 0.12 b	0.60 ± 0.15 b	RI/MS
67	1702	*γ*–hexalactone	0.73 ± 0.19 a	0.24 ± 0.06 c	0.59 ± 0.14 b	–	RI/MS
68	1720	2(3*H*)–Furanone	0.11 ± 0.03 b	0.01 ± 0.00 c	0.16 ± 0.04 a	–	RI/MS
69	1915	*γ*–octalactone	0.16 ± 0.04 c	0.40 ± 0.10 a	0.24 ± 0.06 b	–	RI/MS
70	2012	5–Acetyldihydroxy–2(3*H*)–furanone	0.42 ± 0.11 b	1.02 ± 0.25 a	0.92 ± 0.22 a	–	RI/MS
71	2027	*γ*–Nonalactone	0.11 ± 0.03 a	0.20 ± 0.05 a	0.15 ± 0.04 a	–	RI/MS
		Subtotal	2.00	2.67	2.57	0.60	
		Sulfur-containing compounds (9)					
72	1020	Thiophene	0.25 ± 0.06 a	0.02 ± 0.01 c	0.12 ± 0.04 b	–	RI/MS/S
73	1102	2,3–Dihydroxy–5–methylthiophene	–	–	–	0.05 ± 0.01	RI/MS
74	1106	2,3–Dihydroxythiophene	0.03 ± 0.01 c	0.01 ± 0.01 c	0.07 ± 0.02 b	2.53 ± 0.41 a	RI/MS
75	1213	Diethyl disulfide	0.32 ± 0.07 a	0.04 ± 0.02 b	0.06 ± 0.02 b	–	RI/MS
76	1251	2–Propylthiophene	0.17 ± 0.04 b	0.30 ± 0.13 a	0.39 ± 0.13 a	–	RI/MS
77	1260	Thiazole	0.19 ± 0.04 a	0.02 ± 0.01 c	0.10 ± 0.03 b	–	RI/MS/S
78	1279	4–Methylthiazole	0.07 ± 0.02 b	0.06 ± 0.03 b	0.22 ± 0.07 a	–	RI/MS/S
79	2274	4–Methyl–5–thiazoleethanol	0.13 ± 0.03 b	0.03 ± 0.01 c	0.12 ± 0.04 b	0.44 ± 0.07 a	RI/MS/S
80	2311	1–Dodecanethiol	0.12 ± 0.03 a	0.13 ± 0.05 a	0.03 ± 0.01 b	–	RI/MS
		Subtotal	1.27	0.61	1.12	3.01	
		Nitrogen-containing heterocyclic compounds (8)					
81	1264	Methylpyrazine	0.55 ± 0.13 a	0.49 ± 0.21 a	0.57 ± 0.19 b	–	RI/MS
82	1323	2,5–Dimethylpyrazine	2.74 ± 0.63 a	2.77 ± 1.20 a	2.80 ± 0.93 a	–	RI/MS/S
83	1346	2,3–Dimethylpyrazine	0.28 ± 0.07 b	0.23 ± 0.10 b	0.47 ± 0.16 a	–	RI/MS/S
84	1390	2–Ethyl–6–methylpyrazine	0.93 ± 0.19 a	0.27 ± 0.06 c	0.43 ± 0.09 b	–	RI/MS/S
85	1407	Trimethylpyrazine	1.22 ± 0.25 a	0.72 ± 0.15 b	1.53 ± 0.32 a	–	RI/MS/S
86	1447	3–Ethyl–2,5–dimethylpyrazine	3.64 ± 0.75 b	5.87 ± 1.19 a	5.46 ± 1.14 a	–	RI/MS/S
87	1625	2–Acetyl–3–methylpyrazine	0.25 ± 0.05 b	0.20 ± 0.04 b	0.35 ± 0.07 a	–	RI/MS
88	2412	Indole	0.03 ± 0.01 a	0.05 ± 0.01 a	0.07 ± 0.02 a	–	RI/MS/S
		Subtotal	9.64	10.61	11.68	0.00	
		Oxygen-containing heterocyclic compounds(5)					
89	931	2,5–Dimethylfuran	–	–	–	0.02 ± 0.11	RI/MS
90	1132	2–Pentylfuran	0.45 ± 0.09 b	0.82 ± 0.17 a	0.98 ± 0.21 a	–	RI/MS/S
91	1463	Furfural	–	–	–	–	RI/MS/S
92	1670	Furfuryl alcohol	0.05 ± 0.01 a	0.07 ± 0.02 a	0.06 ± 0.02 a	–	RI/MS
93	2036	2, 5–Dimethyl–4–hydroxy–3(2*H*)–furanone	0.40 ± 0.10 b	0.90 ± 0.25 a	1.07 ± 0.39 a	–	RI/MS
		Subtotal	0.90	1.80	2.11	0.02	
		Others(3)					
94	1972	Phenol	0.08 ± 0.02 b	0.04 ± 0.01 c	0.22 ± 0.08 a	0.09 ± 0.04 b	RI/MS
95	2080	*p*–cresol	0.11 ± 0.03 c	0.49 ± 0.14 b	0.62 ± 0.22 a	0.31 ± 0.07 b	RI/MS/S
96	2129	3–Methylphenol	–	–	–	0.14 ± 0.05	RI/MS/S
		Subtotal	0.18	0.53	0.84	0.53	
		Total	100.00	100.00	100.00	100.00	

^1^ The linear retention indices (RI) detected in the gas chromatography–mass spectrometry (GC–MS) on a DB–WAX column. ^2^ Means ± standard deviations (n = 3), means within the same row with different letters indicate significant differences (*p* < 0.05); “–”, undetected. ^3^ MS, identified by NIST 11 mass spectral database; RI, agreed with the retention indices published in the literature; S, the MS and RI agreed with those of the available authentic chemicals of standards.

**Table 3:** Odor-active compounds identified in the dry-rendered beef fat, using four extraction solvents (pentane, ethyl ether, dichloromethane, and methanol) for solvent-assisted flavor evaporation combined with gas chromatography–olfactometry (SAFE/GC−O).

No.	RIs ^1^	Compounds	Odor Descriptors	NIF% ^2^				Identification Methods ^3^
				Pentane	Dichloromethane	Ethyl Ether	Methanol	
		Aldehydes (26)						
1	881	Butanal	musty, green	60	80	–	–	RI/MS/O/S
2	903	3–Methyl butanal	smoky, malty	100	80	80	20	RI/MS/O/S
3	949	2–Methyl butanal	malty	70	80	20	–	RI/MS/O/S
4	965	Pentanal	green	90	10	20	–	RI/MS/O/S
5	1110	Hexanal	green, grass	100	40	20	–	RI/MS/O/S
6	1123	Heptanal	green, fatty	–	40	80	–	RI/MS/O/S
7	1205	(*E*)–2–Hexenal	green, fatty	90	10	20	–	RI/MS/O/S
8	1252	Octanal	green, citrus	70	40	100	–	RI/MS/O/S
9	1324	(*E*)–2–Octenal	green, fatty	80	80	40	–	RI/MS/O/S
10	1353	(*E*,*Z*)–2,4–Heptadienal	green, fatty	100	10	20	–	RI/MS/O/S
11	1371	Nonanal	citrus, green	80	10	80	–	RI/MS/O/S
12	1396	(*Z*)–Nonenal	green	–	–	40	–	RI/MS/O
13	1408	(*E*)–2–Dodecenal	fatty, paper	–	–	20	–	RI/MS/O/S
14	1461	(*E*,*E*)–2,4–Heptadienal	green, fatty	–	60	–	–	RI/MS/O/S
15	1495	(*E*)–Decanal	fatty	90	10	40	40	RI/MS/O/S
16	1523	(*E*)–2–Nonenal	fatty, paper	–	80	20	–	RI/MS/O/S
17	1527	(*E*,*E*)–2,4–Nonadienal	fatty	–	20	20	40	RI/MS/O/S
18	1532	Benzaldehyde	almond	–	10	–	–	RI/MS/O/S
19	1572	(*E*,*E*)–2,6–Nonadienal	cucumber	100	20	100	–	RI/MS/O/S
20	1630	(*E*)–2–Decenal	fatty	80	20	40	–	RI/MS/O/S
21	1603	(*E*,*E*)–2,4–Octadienal	fatty	–	10	–	–	RI/MS/O/S
22	1727	(*E*)–2–Undecylenal	fatty	–	40	–	–	RI/MS/O/S
23	1787	(*E*,*E*)–2,4–Decadienal	fried fat	–	100	20	40	RI/MS/O/S
24	1811	2–Dodecenal	fatty	90	–	–	60	RI/MS/O/S
25	1826	Tridehydehyde	fatty	–	4	–	–	RI/O
26	1860	12–Methyltridecanal	beef fat–like	50	10	40	–	RI/O/S
		Alcohols (9)						
27	1252	2–Pentanol	alcoholic	100	20	–	–	RI/MS/O/S
28	1546	Octanol	fatty	90	20	–	40	RI/MS/O/S
29	1568	2,3–Butanediol	fruity	–	–	4	–	RI/MS/O/S
30	1845	Phenylethyl alcohol	rose	–	–	4	20	RI/MS/O/S
31	1899	1–Undecanol	fruity	–	–	20	–	RI/MS/O/S
32	1945	1–Dodecanol	wax, sweet	50	10	20	20	RI/MS/O/S
33	1991	(*Z*)–4–Dodecanol	green, sweet	–	–	40	–	RI/MS/O/S
34	2050	1,4–Pentanediol	green, sweet	–	–	20	40	RI/MS/O/S
35	2188	Tetradecyl alcohol	creamy	90	–	–	–	RI/MS/O/S
		Ketones (8)						
36	1074	2,3–Pentanedione	buttery	30	–	–	–	RI/MS/O/S
37	1216	Hexanone	fruity	40	–	–	–	RI/MS/O/S
38	1306	1–Octen–3–one	mushroom	100	20	80	40	RI/MS/O/S
39	1427	3,5–Octanedione	creamy	60	–	–	–	RI//O
40	1527	2–Nonanone	fruity	90	–	–	–	RI/MS/O/S
41	1914	2–Tetradecenone	fatty	70	10	4	20	RI/MS/O/S
42	2219	2–Heptadecanone	fatty	–	–	20	–	RI/MS/O/S
43	2298	*δ*–Undecanone	fruity, fatty	30	–	–	–	RI/O
		Sulfur-containing compounds (11)						
44	749	Dimethyl disulfide	onion, cabbage	50	–	–	–	RI/O/S
45	919	Diethyl disulfide	onion	–	20	–	–	RI/O/S
46	1131	Thiophene	sulfur	90	–	–	–	RI/MS/O/S
47	1320	2–Mercaptothiophene	meaty	–	–	–	20	RI/MS/O/S
48	1385	Dimethyl trisulfide	sulfury, meaty	–	–	–	20	RI/O
49	1410	2–Methyl–3–furanthiol	meaty	–	80	–	–	RI/MS/O/S
50	1438	2–Pentylthiophene	fruity	90	10	20	40	RI/MS/O/S
51	1446	3–Methylthiopropanal	cooked potato	100	80	100	100	RI/MS/O/S
52	1687	4–Methylthiazole	roasted meat	80	100	60	20	RI/MS/O/S
53	1737	2–Acetylthiazoline	roasted meat	100	–	80	–	RI/O/S
54	2286	4–Methyl–5–thiazoleethanol	meaty	–	–	–	20	RI/MS/O/S
		Nitrogen-containing compounds (5)						
55	1338	2,5–Dimethylpyrazine	popcorn, roasted	90	100	–	100	RI/MS/O/S
56	1364	2–Ethyl–6–methylpyrazine	roasted, earthy	–	–	–	20	RI/MS/O/S
57	1419	2–Ethyl–3,5–dimethylpyrazine	roasted	70	100	4	–	RI/MS/O/S
58	1631	2–Acetylpyrazine	roasted	–	–	20	–	RI/MS/O/S
59	2209	3–Methylindole	mothball–like	70	–	–	–	RI/MS/O/S
		Acids (5)						
60	1507	Propionic acid	sour	–	–	–	20	RI/MS/O/S
61	1560	2–Methylpropionic acid	sour	–	–	–	40	RI/MS/O/S
62	1618	4–Hydroxybutyric acid	creamy, beer–like	100	–	–	–	RI/MS/O/S
63	1665	3–Methylbutyric acid	sour	–	–	20	40	RI/MS/O/S
64	1911	Heptanoic acid	cheesy	–	10	–	–	RI/MS/O/S
		Lactones (2)						
65	1899	*γ*–Octalactone	coconunt–like	70	–	–	–	RI/MS/O/S
66	2294	*γ*–Undecalactone	fruity	–	–	20	40	RI/MS/O/S
		Oxygen-containing heterocyclic compound (1)						
67	2018	2,5–Dimethyl–4–hydroxy–3(2*H*)–furanone	caramel	50	100	100	80	RI/MS/O/S
		Others (13)						
68	1268	2–Pentylfuran	green bean	–	20	–	–	RI/MS/O/S
69	1596	Unknown	green, fatty	–	–	–	40	RI
70	1904	Unknown	fatty	–	–	–	20	RI
71	1924	Unknown	fruity	60	–	–	–	RI
72	1986	Unknown	metallic	40	–	–	–	RI
73	2061	*p*–Cresol	phenolic	100	20	100	40	RI/MS/O/S
74	2099	*o*–Cresol	musty, phenolic	60	–	–	–	RI/MS/O/S
75	2147	3–Methyl phenol	smoky, phenolic	–	–	20	–	RI/MS/O/S
76	2165	4–Ethylphenol	phenolic	–	20	–	–	RI/MS/O/S
77	2200	Vanillin	caramel	–	–	–	60	RI/MS/O/S
78	2276	Unknown	jujube	10	–	–	–	RI
79	2285	Unknown	fruity	20	–	–	–	RI
80	2293	Unknown	grass	50	–	–	–	RI

^1^ The linear retention indices (RIs) sniffed in the GC−O analysis on a DB–WAX column. ^2^ Means of the NIF values (%), that is, percentages of the summed frequencies of a compound perceived by all panelists divided by the total times of a sample analyzed (n = 3), “–”, not detected. ^3^ MS, based on GC–MS identification; RI, agreed with the retention indices published in the literature; O, agreed with the odor descriptors published in the literature; S, all the analytical parameters (RI, MS, and O) agreed with the available authentic chemicals of standards.

**Table 4:** Scanned ions, diluted concentrations of the standards, and the obtained calibration curves during quantitation of the odorants by gas chromatography–mass spectrometry (GC–MS) in selected ion monitoring (SIM) detection.

RIs ^1^	Compounds	Ions (*m*/*z*)	Calibration Curves	Concentrations (μg/mL)
919	Dimethyl disulfide	94, 79	*y* = 3.803*x* + 0.0485; *R^2^ =* 0.9735	0.10–100
1071	Hexanal	82, 72	*y* = 31.203*x* − 11.398; *R^2^ =* 0.9963	100–1200
1209	Heptanal	81, 70	*y* = 1.775*x* − 0.772; *R^2^ =* 0.9879	20–150
1268	2–Pentylfuran	138, 81	*y* = 1.008*x* − 0.264; *R^2^ =* 0.9967	1–100
1291	Octanal	110, 84	*y* = 11.771*x* + 2.671; *R^2^ =* 0.9747	50–200
1304	1–Octen–3–one	72, 57	*y* = 93.409*x* − 2.308; *R^2^ =* 0.9976	5–150
1331	2–Ethyl–3,5–dimethylpyrazine	135, 108	*y* = 4.149*x* − 0.140; *R^2^ =* 0.9983	1.0–20
1390	Nonanal	114, 98	*y* = 1.523*x* − 0.3119; *R^2^* = *0.9924*	20–100
1410	2–Methyl–3–furanthiol	114, 85	*y* = 109.560*x* + 0.012; *R^2^ =* 0.9998	40–250
1437	(*E*)–2–Octenal	97, 83	*y* = 9.9092*x* − 0.425; *R^2^ =* 0.9979	50–200
1441	3–Methylthiopropanal	104, 76	*y* = 1.511*x* − 0.919; *R^2^ =* 0.9908	50–150
1478	Decanal	112, 95	*y* = 1.512*x* − 0.996; *R^2^ =* 0.9909	10–50
1523	(*E*)–2–Nonenal	96, 83	*y* = 15.163*x* − 1.300; *R^2^ =* 0.9953	20–50
1532	Benzaldehyde	106, 77	*y* = 1.030*x* − 0.220; *R^2^ =* 0.9969	5.0–20
1540	(*E*,*E*)–2,4–Nonadienal	120, 70	*y* = 141.130*x* + 0.112; *R^2^ =* 0.9997	10–100
1582	Octanol	84, 70	*y* = 1.519*x* − 0.531; *R^2^ =* 0.9881	20–50
1600	(*E*)–2–Decenal	136, 110	*y* = 1.294*x* + 0.576; *R^2^ =* 0.9694	1–100
1603	(*E*,*E*)–2,4–Octadienal	124, 81	*y* = 1.487*x* − 1.164; *R^2^ =* 0.9959	0.1–5.0
1625	(*E*,*E*)–2,4–Decadienal	152, 81	*y* = 15.098*x* − 0.538; *R^2^ =* 0.9837	50–300
1980	12–Methyltridecanal	109, 95	*y* = 12.949*x* + 0.101; *R^2^ =* 0.9926	0.5–10
2053	*p*–Cresol	107, 90	*y* = 14.944*x* − 0.581; *R^2^ =* 0.9945	10–50
2165	4–Ethylphenol	122, 107	*y* = 1.515*x* − 0.888; *R^2^ =* 0.9895	0.2–5.0
1489	1,2–Dichlorobenzene	114, 111	(Internal standard)	30

^1^ The linear retention indices (RI) detected on a DB–WAX column in the GC–MS analysis.

**Table 5:** Concentrations and odor-active values (OAVs) for the odorants in the dry-rendered beef fat using pentane and dichloromethane extraction for solvent-assisted flavor evaporation (SAFE) and selected ion monitoring (SIM) detection of gas chromatography–mass spectrometry (GC–MS) for quantitative analysis.

RIs ^1^	Compounds	Amounts (ng/g) ^2^		OAVs ^3^		
		Dichloromethane	Pentane	Dichloromethane	Pentane	Thresholds ^4^ (ng/g)
919	Dimethyl disulfide	0.71 ± 0.11 b	1.81 ± 0.27 a	–	–	12
1071	Hexanal	102.40 ± 1.34 a	15.82 ± 11.43 b	1	–	75
1209	Heptanal	55.47 ± 1.09 a	20.03 ± 1.07 b	–	–	230
1268	2–Pentylfuran	11.63 ± 1.74 a	2.88 ± 0.15 b	2	–	5
1291	Octanal	64.54 ± 1.65 b	77.89 ± 1.24 a	1	1	56
1306	1–Octen–3–one	10.64 ± 0.29 a	5.16 ± 0.80 b	5	3	2
1331	2–Ethyl–3,5–dimethylpyrazine	3.30 ± 0.19 a	1.68 ± 0.08 b	1	1	3
1390	Nonanal	–	10.84 ± 1.50	–	–	1000
1410	2–Methyl–3–furanthiol	21.62 ± 1.16 b	48.63 ± 2.15 a	2162	4863	0.01
1437	(*E*)–2–Octenal	56.84 ± 1.54 a	54.89 ± 2.89 a	19	18	3
1441	3–Methylthiopropanal	56.56 ± 3.83 a	6.14 ± 0.26 b	283	31	0.2
1478	Decanal	22.11 ± 1.34 b	43.06 ± 4.89 a	–	–	650
1523	(*E*)–2–Nonenal	48.87 ± 0.95 b	56.49 ± 42.29 a	1	1	45
1532	Benzaldehyde	7.64 ± 1.26 a	5.65 ± 4.24 a	–	–	60
1540	(*E*,*E*)–2,4–Nonadienal	23.60 ± 1.14 a	8.40 ± 0.83 b	16	6	1.5
1582	Octanol	28.44 ± 0.75 b	78.33 ± 7.59 a	1	3	27
1600	(*E*)–2–Decenal	2.97 ± 0.32 b	60.93 ± 11.80 a	–	6	10
1603	(*E*,*E*)–2,4–Octadienal	0.99 ± 0.05 b	3.49 ± 0.31 a	–	–	1000
1625	(*E*,*E*)–2,4–Decadienal	175.12 ± 1.43 a	32.90 ± 4.56 b	1	–	180
1980	12–Methyltridecanal	0.74 ± 0.08 b	2.55 ± 0.40 a	7	26	0.1
2053	*p*–cresol	29.54 ± 3.22 a	1.22 ± 0.09 b	1	–	25
2165	4–Ethylphenol	0.24 ± 0.06	–	–	–	/

^1^ Retention indices (RIs) on a DB–WAX column; ^2^ Means “±” standard derivations (n = 3); means within the same row with different letters indicate significant differences (*p* < 0.05). ^3^ odor-active values (OAVs) derived by concentrations of compounds divided by odor thresholds. ^4^ The detection odor thresholds in oils, referred to Gemert, L. J. V., Odour Thresholds: Compilations of Flavour Threshold Values in Air, Water and Other Media, Second enlarged and revised edition; Oliemans Punter & Partners BV: Utrecht, the Netherlands, 2011.

## Data Availability

All relevant data are included in the article.
